# Development and validation of a multivariate nomogram for predicting retinopathy of prematurity in infants with gestational age ≤34 weeks

**DOI:** 10.3389/fped.2025.1576979

**Published:** 2025-05-09

**Authors:** Leilei Shen, Ruixue Zheng, Xiaodong Sun, Sheng Chen

**Affiliations:** Department of Pediatrics, Third Military Medical University Southwest Hospital, Chongqing, China

**Keywords:** premature infants, retinopathy of prematurity, risk factors, predictive nomogram, neonatal health

## Abstract

**Purpose:**

To delineate risk factors and develop a predictive nomogram for retinopathy of prematurity (ROP) in infants with gestational age (GA) ≤34 weeks.

**Methods:**

We conducted a comprehensive retrospective analysis of infants with GA ≤34 weeks, divided into ROP and non-ROP groups based on fundus screening results. Clinical and laboratory data were collected to identify risk factors associated with ROP. Multivariable logistic regression was performed to identify independent predictors, and a nomogram was developed to predict the occurrence of ROP in infants with GA ≤34 weeks.

**Results:**

Our analysis identified five independent risk factors for ROP in infants with GA ≤34 weeks: hypertensive disorders of pregnancy (HDP), number of blood transfusions, oxygen therapy time (OTT), oxygen therapy concentration (OTC) >50%, and blood glucose spikes in the first postnatal week. These predictors were incorporated into a nomogram to estimate individual ROP risk. The predictive model achieved a C-index of 0.923 (95% CI: 0.888–0.959), indicating high predictive accuracy. Internal validation of the nomogram demonstrated excellent calibration and practical utility for clinical decision-making.

**Conclusions:**

The established predictive model, incorporating five key clinical parameters, offers clinicians a practical instrument to stratify ROP risk in neonates born at ≤34 weeks’ gestation. This clinical tool demonstrates significant utility in guiding intervention protocols, potentially enhancing patient outcomes through early identification and optimized management strategies.

**Registration number:**

ChiCTR2400086213.

## 1 Introduction

Retinopathy of prematurity (ROP) is a devastating neurovascular disease of the retina in newborn infants that can lead to visual defects or even blindness, accounting for approximately 6%–8% of the causes of blindness in children (1). With advances in perinatal medicine and neonatology and the widespread establishment of neonatal intensive care units (NICUs), survival rates for preterm and low-birth-weight infants have increased significantly. As a result, the number of infants at risk for ROP has increased.

The incidence of ROP varies widely across different countries and is linked to the socioeconomic development as well as the quality and accessibility of health care facilities (2). In low and middle-income countries an “epidemic” of ROP blindness is currently occurring. In 2010, ten countries including China accounted for nearly two- thirds of all cases of visual impairment due to ROP (3). Recent data indicate that the incidence of ROP in low birth weight infants in China ranges from 8.2% to 17.8%, with the incidence in very premature infants as high as 65.1% (4–7).

Despite extensive research efforts, a comprehensive understanding of the risk factors for ROP remains incomplete. To address this critical gap, we conducted a retrospective analysis of 452 preterm infants with a gestational age (GA) of ≤34 weeks. The objective of our study was to elucidate the associations between clinical characteristics, laboratory parameters, and the incidence of ROP. We constructed and validated robust predictive models to enhance early detection and improve outcomes in this vulnerable population.

## 2 Material and methods

### 2.1 Study subjects

We performed a retrospective analysis of clinical records of infants with GA ≤34 weeks who were admitted to our NICUs immediately after birth, from January 2018 to January 2024. Inclusion criteria: (1) Premature infants with GA ≤34 weeks; (2) Informed consent of the parents for fundus screening; (3) Complete clinical information was available. Diagnostic classification: ROP group: All infants meeting any stage of ROP according to the International Classification of Retinopathy of Prematurity (ICROP-3) (1), including: Stage 1–5 retinopathy (Zone I–III); With or without plus disease; Requiring laser/surgery (Type 1 ROP) or observation (Type 2 ROP); Non-ROP group: Infants with complete retinal vascularization (Zone III) confirmed by serial examinations, or immature retina without neovascular changes. Exclusion criteria: (1) Congenital eye diseases such as retinoblastoma, congenital cataract, glaucoma, etc.; (2) Hereditary metabolic diseases and severe congenital malformations; (3) Incomplete clinical data; (4) Failure to complete the fundus examination.

This study was approved by the Ethics Committee (KY2024171) and adhered to the tenets of the Declaration of Helsinki. All data were completely anonymized to ensure patient confidentiality.

### 2.2 Screening criteria

According to the “Chinese Guidelines for Screening Retinopathy of Prematurity (2014)” (8), the initial screening for fundus lesions was performed at 4–6 weeks after birth or at 31–32 weeks of corrected GA. The scope of the screening included the peripheral retinal blood vessels to ensure comprehensive lesion detection.

### 2.3 Relevant definitions

Relevant definitions: (1) The total count of blood product transfusions, encompassing red cells, plasma, platelets, and other components; (2) oxygen therapy time (OTT): The cumulative duration of oxygen therapy, including mechanical ventilation, hooded oxygen therapy, and nasal cannula oxygen therapy; (3) Oxygen therapy concentration (OTC) >50%: The concentration of oxygen used during therapy when it exceeds 50%; (4) Hyperglycemia (<1 week): In the first postnatal week, if the blood glucose level is ≥7.0 mmol/L, a re-measurement of peripheral blood glucose on the contralateral foot is conducted. Hyperglycemia is diagnosed if the level remains ≥7.0 mmol/L; (5) Glucose Monitoring: All subjects underwent routine pre-feeding glucose monitoring four times daily during the first week of life. The frequency was increased to 8–24 times per day upon detection of abnormal glucose levels. Monitoring frequency was gradually reduced after two consecutive normal readings, returning to four times per day; (6) Blood sugar spikes (<1 week): The peak blood glucose value recorded during the first postnatal week; (7) Average blood sugar (<1 week): The mean blood glucose level during the first postnatal week, calculated by dividing the sum of all monitored values by the total number of monitoring sessions.

### 2.4 Data collection

Basic neonatal demographic information was collected from the medical records, including gender, gestational age, birth weight, mode of delivery, maternal gestational comorbidities, and prenatal and intrapartum conditions. Additionally, data on comorbidities, blood glucose levels in the first postnatal week, and details of oxygen therapy were collected. For neonates in the ROP group who required therapeutic interventions, all clinical data were systematically collected prior to treatment initiation—specifically at the time of ROP diagnosis. This design ensures that the captured variables reflect pre-intervention baseline characteristics, thereby preserving the integrity of risk factor associations with ROP development. By anchoring data collection to the diagnostic timepoint, we mitigate potential confounding effects of post-treatment physiological changes on predictor variables.

### 2.5 Development and assessment of the model

Potential predictors were first identified by univariate analysis. Significant variables were then included in multivariate logistic regression analyses to develop a prediction model for ROP in infants with GA ≤34 weeks. Subsequently, internal verification was conducted to create a nomogram with excellent calibration and discrimination capabilities. In the multivariate analysis, variables with *P* < 0.05 were included in the nomogram. The foundation of the nomograph lies in scaling each regression coefficient in multiple logistic regression to a range of 0–100 points. The cumulative score, representing the predictive probability, can be obtained by summing up the scores assigned to each variable. The prediction accuracy and consistency of the model are assessed using the calibration curve, receiver operating characteristic (ROC) curve, the area under the ROC curve (AUC), consistency index (C index), and the sensitivity, specificity, positive predictive value (PPV), negative predictive value (NPV) were also assessed. The net benefits of the model to neonates are reflected by decision curve analysis (DCA). By bootstrapping 1,000 resamples, identification and calibration are assessed.

### 2.6 Statistical analysis

This study uses ROP as the dependent variable to construct a prediction model, takes the AUC value of the prediction model as the main index, and utilizes PASS 15 software (NCSS, Kaysville, Utah, USA) to calculate the Power of the current sample size. Finally, this study included 65 cases of ROP and 387 cases of non-ROP. The AUC value of the prediction model was 0.923. Under the condition of setting a two-sided test *α* = 0.05, we inputted data into PASS 15 software (NCSS, Kaysville, Utah, USA) for Curve Tests and obtained a Power >0.999.

All statistical analyses were determined using SPSS software version 26.0 (IBM SPSS Statistics, Chicago, IL, USA) and R software (Version 4.1.2). Continuous variables are expressed as mean ± standard deviation. Non-parametric data are expressed as median (25%–75% interquartile range). Categorical variables are presented as absolute numbers and percentages. Continuous data between two groups were compared by independent samples *t*-test or Mann–Whitney *U*-test. Categorical data between two groups were compared by Chi-square test. According to the results of logistic multivariate regression analysis, a predictive model was constructed, and receiver operator characteristic (ROC) curve analysis was used to evaluate the area under the curve (AUC) and its 95% CI, and the model was validated by random sampling 1,000 times using the bootstrap method. Lastly, a calibration plot was constructed. The performance of the predictive model was evaluated by the Hosmer–Lemeshow test, AUC, and goodness-of-fit. Decision curve analysis (DCA) was used to validate the clinical net benefit rate of the predictive model.

## 3 Results

### 3.1 Risk factors for ROP in infants with GA ≤34 weeks

**Figure 1:**
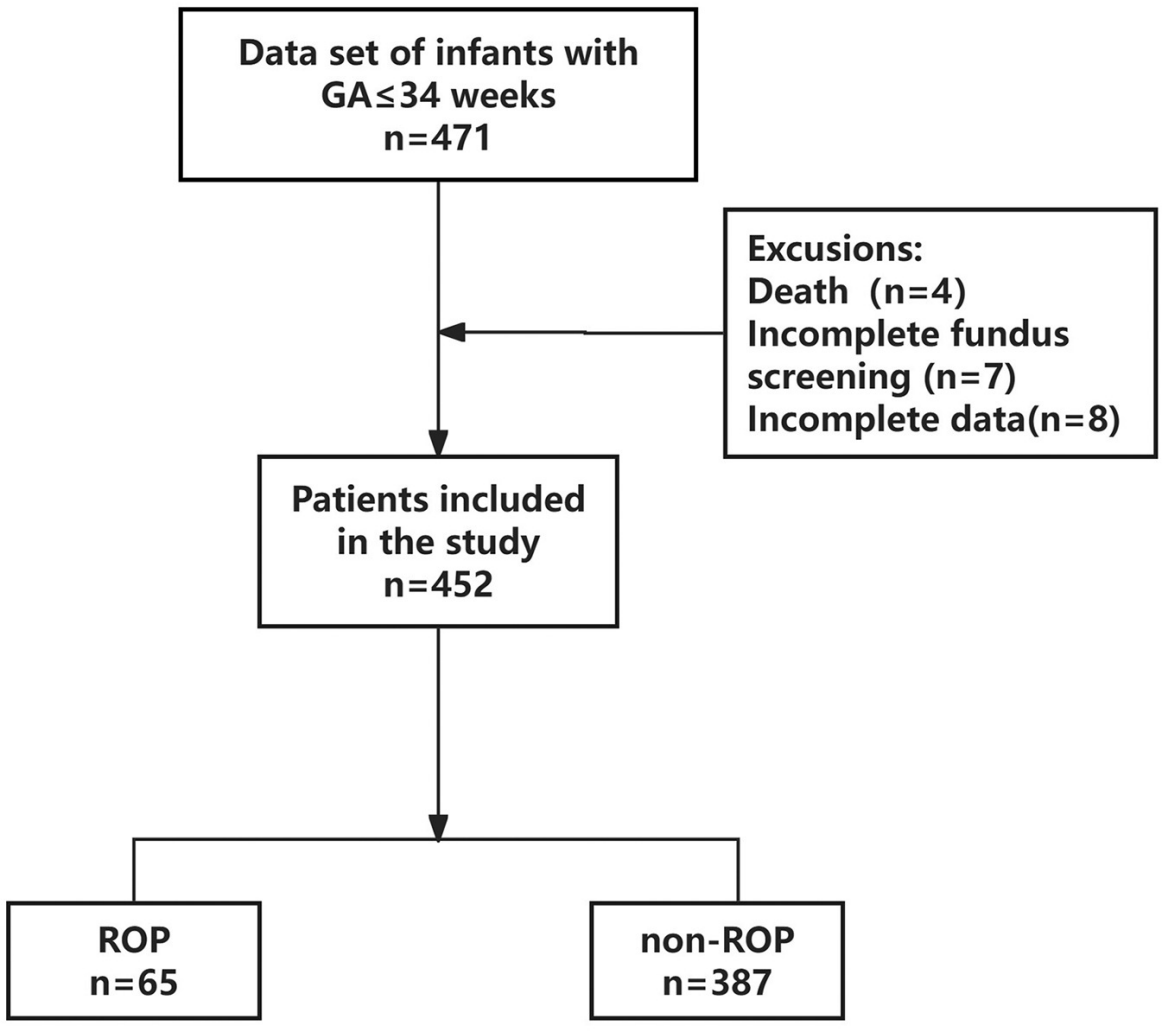
Flow chart for patient selection. GA, gestational age; ROP, retinopathy of prematurity.

A total of 471 patients’ clinical information was obtained; 19 cases did not meet the inclusion criteria, of which 4 died during hospitalization, 7 did not complete fundus screening, and 8 had incomplete clinical data. Finally, 452 patients were enrolled in this study (Figure 1). The differences in all data between the ROP and non-ROP group were shown in Table 1. ROP group had significant differences in GA, birth weight, hypertensive disorders of pregnancy (HDP), and fetal distress. In addition, comorbidities and treatments including bronchopulmonary dysplasia (BPD), sepsis, intracranial hemorrhage (ICH), respiratory distress syndrome (RDS), necrotizing enterocolitis (NEC), number of blood transfusions, OTT, OTC >50%, parenteral nutrition (PN) >14 days (d), hyperglycemia (<1 week), blood sugar spikes (<1 week), and the average blood sugar (<1 week) showed a significant difference between ROP and non-ROP patients (*P* < 0.05).

**Table 1:** Comparison of baseline clinical characteristics comorbidities, and treatments between the non-ROP group and ROP group.

Variables	non-ROP group (*n* = 387)	ROP group (*n* = 65)	*P*
General data			
Gender, male, *n* (%)	187 (48.3)	33 (50.8)	0.715
Gestational age (weeks)	33.00 (31.10, 33.50)	30.2 (28.80, 32.25)	<0.001
Birth weight (kg)	1.72 ± 0.41	1.42 ± 0.40	<0.001
Delivery mode, eutocia, *n* (%)	79 (20.4)	20 (30.8)	0.062
Pregnancy complication			
HDP, *n* (%)	67 (17.3)	36 (55.4)	<0.001
GDM, *n* (%)	121 (31.3)	22 (33.8)	0.679
ICP, *n* (%)	40 (10.3)	6 (9.2)	0.785
Perinatal condition			
ACT, *n* (%)	209 (54.0)	36 (55.4)	0.836
Fetal distress, *n* (%)	62 (16.0)	26 (40.0)	<0.001
Comorbidities			
BPD, *n* (%)	74 (19.1)	39 (60.0)	<0.001
Sepsis, *n* (%)	17 (4.4)	15 (23.1)	<0.001
ICH, *n* (%)	37 (9.6)	16 (24.6)	<0.001
PDA, *n* (%)	289 (74.7)	50 (76.9)	0.699
RDS, *n* (%)	170 (43.9)	56 (86.2)	<0.001
NEC, *n* (%)	20 (5.2)	9 (13.8)	0.018
Hyperglycemia (<1 week), *n* (%)	34 (8.8)	37 (56.9)	<0.001
Laboratory metrics			
Blood sugar spikes (<1 week) (mmol/L)	5.50 (4.90, 6.30)	8.20 (5.60, 8.55)	<0.001
Average blood sugar (<1 week) (mmol/L)	4.30 (3.90, 4.70)	5.10 (4.50, 6.10)	<0.001
Treatments			
Number of blood transfusions (*n*)	0.00 (0.00, 3.00)	6.00 (2.5, 12.50)	<0.001
OTT (days)	7.00 (2.00, 22.00)	37.00 (7.5.00, 54.50)	<0.001
OTC >50%, *n* (%)	25 (6.5)	35 (53.8)	<0.001
PN >14 days, *n* (%)	136 (35.1)	49 (75.4)	<0.001

HDP, hypertensive disorders of pregnancy; GDM, gestational diabetes mellitus; ICP, intrahepatic cholestasis of pregnancy; ACT, antenatal corticosteroid therapy; BPD, bronchopulmonary dysplasia; ICH, intracranial hemorrhage; PDA, patent ductus arteriosus; RDS, respiratory distress syndrome; NEC, necrotizing enterocolitis; OTT, oxygen therapy time; OTC, oxygen therapy concentration.

### 3.2 Screening for predictive factors

Multivariable logistic regression analysis showed that five factors were independent predictors of ROP in infants with GA ≤34 weeks, as follows: HDP [*P* = 0.001, odds ratio (OR) 3.777, 95% confidence interval (CI) 1.766–8.077], number of blood transfusions (*P* < 0.001, OR 1.215, 95% CI 1.127–1.311), OTT (*P* = 0.002, OR 1.031, 95% CI 1.012–1.051), OTC >50% (*P* < 0.001, OR 5.550, 95% CI 2.413–12.766), and blood sugar spikes (<1 week) (*P* = 0.025, OR 1.288, 95% CI 1.032–1.609) (Table 2).

**Table 2:** Predictors of ROP in infants with GA ≤34 weeks.

Variables	B	SE	Wald*χ*^2^	*P*	OR (95% CI)
HDP	1.329	0.388	11.739	0.001	3.777 (1.766–8.077)
Blood transfusions	0.195	0.039	25.486	<0.001	1.215 (1.127–1.311)
OTT	0.031	0.010	9.855	0.002	1.031 (1.012–1.051)
OTC >50%	1.714	0.425	16.262	<0.001	5.550 (2.413–12.766)
Blood sugar spikes (<1 week)	0.253	0.113	4.998	0.025	1.288 (1.032–1.609)
Constants	−5.865	0.766	58.583	<0.001	–

HDP, hypertensive disorders of pregnancy; OTT, oxygen therapy time; OTC, oxygen therapy concentration.

The final regression model, as shown in Table 2, can be represented by the formula Ln(P/1-P) = 1.329*HDP (yes = 1, no = 0) + 0.195*blood transfusions + 0.031*OTT + 1.714*OTC (>50%=1, ≤ 50%=0) + 0.253 * blood sugar spikes-5.865.

### 3.3 Risk prediction nomogram development

**Figure 2:**
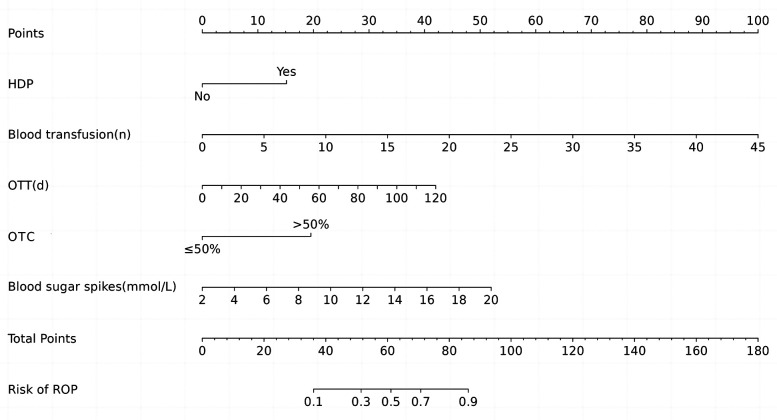
Nomogram of ROP in infants with GA ≤34 weeks. HDP, hypertensive disorders of pregnancy; OTT, oxygen therapy time; OTC, oxygen therapy concentration.

According to the results of multivariable logistic regression analysis, the following factors were associated with ROP: HDP, number of blood transfusions, OTT, OTC >50% and blood sugar spikes (<1 week). These five factors were included in the prediction model, and a nomogram was created to visualize the results of the regression analysis (Figure 2).

### 3.4 Validation of nomogram

**Figure 3:**
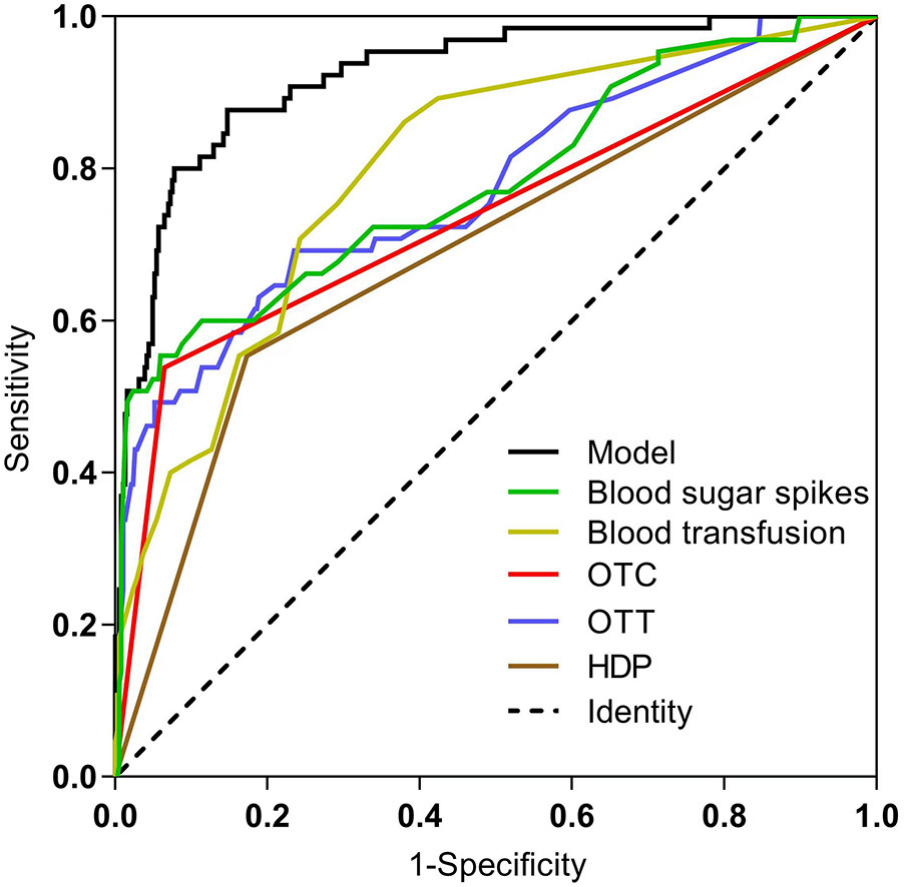
Comparison of ROC curves for each predictor and prediction model. HDP, hypertensive disorders of pregnancy; OTT, oxygen therapy time; OTC, oxygen therapy concentration.

The ROC curves and corresponding AUC values generated by HDP, number of blood transfusions, OTT, OTC >50%, blood sugar spikes (<1 week) and prediction model are 0.690, 0.799, 0.776, 0.737, 0.782 and 0.923 respectively, as shown in Table 3 and Figure 3. The cut-off values for HDP, number of blood transfusions, OTT, OTC >50%, blood sugar spikes (<1 week) and predictive modeling were obtained by using the maximum value of Jorden's index as the optimal threshold, and the associated sensitivity, specificity, positive predictive value and negative predictive value are shown in Table 3. The difference in AUC values between the prediction model and each independent predictor is statistically significant (*P* < 0.05).

**Table 3:** Comparison of the prediction effect of each independent predictor and prediction model of ROP.

Variables	AUC	95% CI	*P*	Cutoff values	Sensitivity	Specificity	PPV	NPV
HDP	0.690	0.614–0.766	<0.001	–	55.4	82.7	35.0	91.7
Blood transfusions	0.799	0.741–0.857	<0.001	1.5	86.2	62.0	27.6	96.4
OTT	0.776	0.708–0.844	<0.001	24.5	69.2	76.5	33.1	93.7
OTC >50%	0.737	0.659–0.814	<0.001	–	53.8	93.5	58.3	92.3
Blood sugar spikes (<1 week)	0.782	0.713–0.851	<0.001	7.45	55.4	94.1	61.0	92.6
Predictive model	0.923	0.888–0.959	<0.001	0.11698	87.7	85.3	50.0	97.6

HDP, hypertensive disorders of pregnancy; OTT, oxygen therapy time; OTC, oxygen therapy concentration.

**Figure 4:**
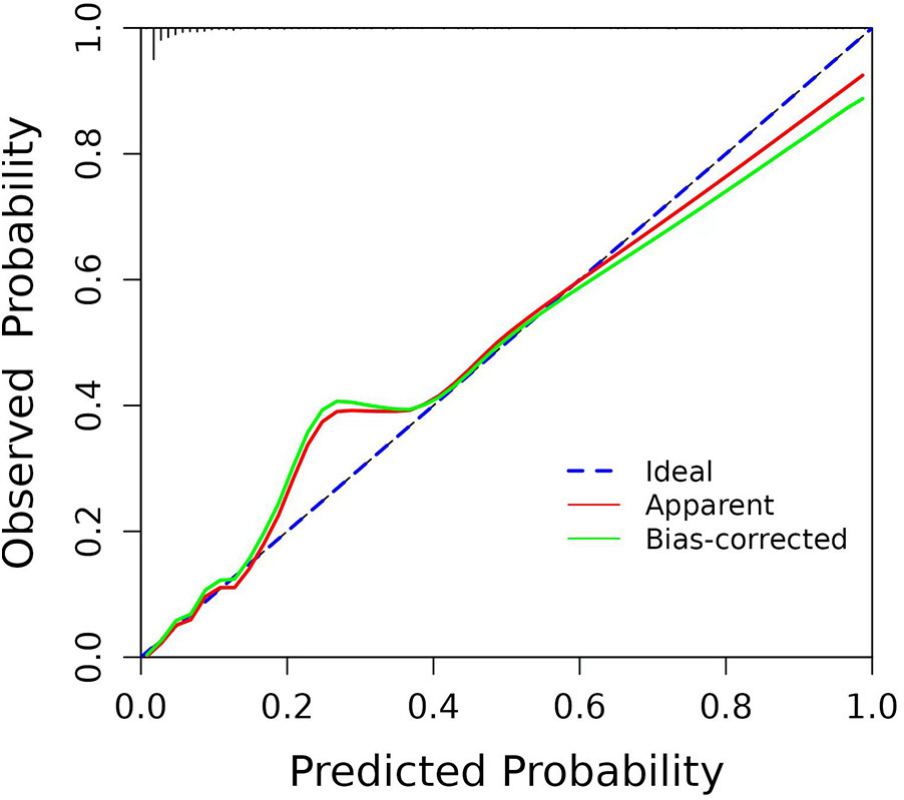
Calibration curve for predicting the probability of ROP in infants with GA ≤34 weeks.

By internally validating the accuracy of the prediction model using the Bootstrap resampling technique, and the Hosmer-Lemeshow test showed that χ*^2^* = 7.715, *P* = 0.462 > 0.05, the C-index was 0.923, with a strong fit between the original and corrected curves, demonstrating the effectiveness of the prediction model (Figure 4).

### 3.5 Net benefit of the nomogram

**Figure 5:**
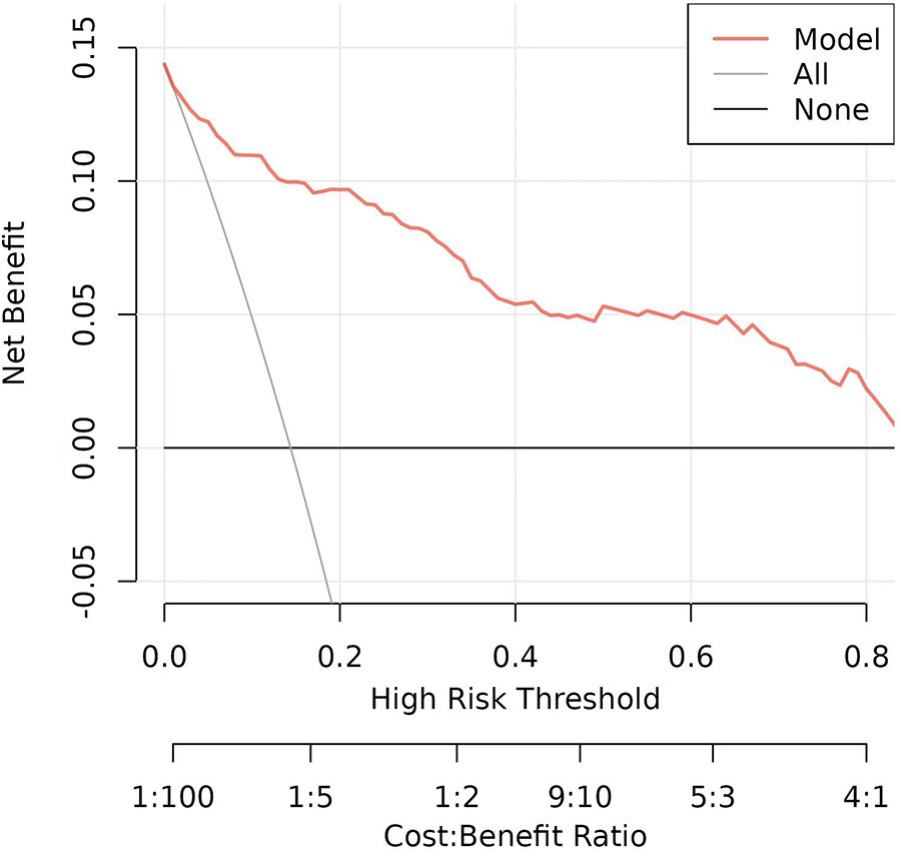
Nomogram decision curve for ROP in infants with GA ≤34 weeks.

Decision analysis (DCA) was performed on the data to evaluate the clinical utility of the prediction model. The analysis of the decision curve analysis that the model can significantly improve the clinical efficiency in predicting ROP in infants with GA ≤34 weeks, as shown in Figure 5.

## 4 Discussion

ROP, responsible for the majority of visual sequelae in premature infants, is one of the main preventable causes of childhood blindness (9). Currently, the ROP screening guidelines for preterm infants vary between countries, especially between developed and developing countries (10–13). We selected preterm infants with GA ≤34 weeks as study subjects in the hope that the resulting predictive model would ensure that infants who are likely to require treatment are not missed. We included all stages of ROP (Type 1 and Type 2) to enhance the sensitivity of screening. This approach helps to avoid missed diagnoses of early-stage lesions that may continue to progress after discharge. This study showed that HDP, number of blood transfusions, OTT, OTC >50% and blood sugar spikes (<1 week) were predictors of ROP in infants with GA ≤34 weeks.

As we know, HDP was strongly associated with certain adverse outcomes in newborns (i.e., preterm birth, small for gestational age, restricted growth and development and intrauterine distress) (14, 15). Nawsherwan et al. (16) reported that HDP was associated with a higher risk of C-section, preterm birth, perinatal mortality, and low birth weight (LBW) in both singleton and twin pregnancies compared with the non-HDP. Neonates born to mothers with HDP had significantly lower GA, mean birth weight, and birth percentile, and the incidence of very premature preterm birth increased by 4.7% (17). A study in two southern provinces China (18) found that the incidence rates of LBW/small-for-gestational-age (SGA) in gestational hypertension and pre-eclampsia group increased by 1.47%/1.9% and 3.86%/4.93% respectively. Our study also indicated that HDP was an independent predictor of ROP and was included in the predictive model. This result is not difficult to understand, vascular endothelial growth factor (VEGF) is currently recognized as an important mechanism of pathological neovascular proliferation in ROP (19), HDP can lead to decreased expression of VEGF antagonist receptors (20), and elevated VEGF expression in the maternal environment ultimately interferes with fetal retinal vascular development, making them more susceptible to ROP after birth. Therefore, regular examinations during pregnancy to monitor the mother's blood pressure status, and active control of blood pressure during pregnancy are important measures to prevent the occurrence of ROP.

A European multicenter study demonstrated that unrestricted threshold blood transfusions are associated with a higher incidence of ROP in very low birth weight infants (21). A meta-analysis identified blood transfusion as an independent risk factor for ROP development in preterm infants with a gestational age (GA) of less than 32 weeks, findings that align with our study results (22). Blood transfusion progressively decreases fetal hemoglobin (HbF) levels in neonates while alleviating anemia. This reduction in HbF impairs retinal perfusion and antioxidant capacity (23). Moreover, blood transfusion increases free iron levels in plasma, which catalyzes the formation of reactive oxygen species and oxygen free radicals (24), thereby elevating the risk of retinal damage. In our study, we specifically evaluated the total count of all blood product transfusions (including red cells, plasma, and platelets) rather than solely red blood cell transfusions, as transfusion of non-erythrocyte products (e.g., platelets, plasma) may reflect more severe clinical conditions (such as sepsis or coagulation disorders) that independently increase ROP risk.

In our study, OTT and OTC >50% were also independent predictors of ROP in infants with GA ≤34 weeks. The longer the oxygen inhalation time, the higher the points in the nomogram model, and the higher the probability of ROP. Retinal hyperoxygenation is a recognized factor in the development of ROP. Premature and low birth weight infants with immature lung development usually require various modalities of oxygen therapy. The high concentration of oxygen can be toxic to the immature retina, inhibiting the development of retinal vasculature, leading to endothelial damage in retinal blood vessels, causing ischemic retinopathy in the avascular zone, and promoting proliferation and constriction of fibro-neovascular membranes, which induce ROP (25). Selection of higher oxygen saturation targets early in clinical care can result in preterm infants being exposed to hyperoxic risks such as fluctuating partial pressures of oxygen, high oxygen concentrations, etc., and is associated with an increased incidence of ROP (26). The United Kingdom's National Institute for Health and Care Excellence (NICE) guidelines recommend a target oxygen saturation of 91% to 95% in preterm infants born at less than 32 weeks of gestation (27). Shukla et al. (28) found that compared with static oxygen standards, biphasic oxygen targets are associated with decreased incidence and severity of ROP without increasing mortality, but the set point for oxygen saturation is currently controversial, and there are no large-scale studies to clarify whether this strategy is helpful in reducing the incidence of ROP and the mortality rate of preterm infants. However, in clinical practice, the indications for oxygen inhalation in premature infants should be strictly controlled to reduce the unregulated use of oxygen. Additionally, close monitoring of oxygen partial pressure and oxygen saturation is necessary to minimize the risk of ROP.

High peak blood glucose in the first postnatal week was also an independent predictor of ROP and was included in our predictive model. It has been found that very low birth weight infant (VLBWI) are prone to hyperglycemia in the first postnatal week (29), and hyperglycemia is usually one of the clinical manifestations of a variety of acute stresses and serious illnesses, as well as early hyperglycemia has been associated with an increased incidence of a variety of complications in VLBWI, including ROP (30). Hyperglycemia in preterm infants can lead to low levels of insulin-like growth factor 1, which is a cytokine necessary for neovascularization formation in the retina (31). Our study showed that the ROP group had higher average blood glucose value in the first postnatal week compared with the non-ROP group. Although the average blood glucose value in the ROP group was within the normal range, the higher average blood glucose could laterally reflect the high number of hyperglycemia exposures or the high glucose level in that time period. In addition, multifactorial analysis showed that high peak blood glucose in the first postnatal week was an independent risk factor for ROP in preterm infants, This may be related to the effect of high glucose concentration on retinal development in preterm infants on the one hand (32, 33), and to the fact that the infants in this group had a younger gestational age, lower body weight, and were relatively sicker on the other hand. Therefore, attention should be paid to blood glucose management during the first week of life, and early hyperglycemia requires close monitoring and timely intervention to prevent exposure to higher glucose concentrations and reduce the occurrence of ROP.

In this investigation, we analyzed clinical and laboratory data from infants with a gestational age (GA) of ≤34 weeks, developed an early risk prediction model for ROP, and validated its high accuracy, reliability, and clinical utility. This model provides clinicians with a practical and user-friendly tool for predicting ROP risk. While our single-center study provides valuable insights, the findings may not be fully generalizable to broader neonatal populations due to inherent limitations in study design and population characteristics. To enhance the robustness and applicability of our predictive model, future research should include multicenter studies with a more diverse neonatal population to validate and refine the predictive factors. Additionally, external validation of the model is essential to confirm its predictive performance and ensure its suitability for clinical practice across various settings.

## 5 Conclusion

In summary, our research indicates that HDP, number of blood transfusions, OTT, OTC exceeding 50%, and blood sugar spikes within the first week of life are significant predictors of ROP in infants with a GA of ≤34 weeks.

## 6 Future directions

To enhance the clinical applicability of our nomogram, future studies will focus on multicenter external validation to evaluate its generalizability across diverse populations and clinical settings, followed by rigorous optimization of risk stratification thresholds using decision-curve analysis and Youden's index to establish clinically actionable cutoffs. These efforts will be complemented by pilot implementation studies in NICUs to assess real-world utility and iterative model refinement incorporating emerging biomarkers and treatment trends, ultimately aiming to develop a dynamic, evidence-based tool that bridges predictive accuracy with practical clinical decision-making for ROP management.

## Data Availability

The original contributions presented in the study are included in the article/Supplementary Material, further inquiries can be directed to the corresponding author.
